# Eclipsed Functional Mitral Regurgitation Destabilizing Hypertrophic Cardiomyopathy: An Unusual Case Treated With MitraClip

**DOI:** 10.1016/j.cjco.2020.12.010

**Published:** 2020-12-15

**Authors:** Catherine Bourque, Marina Dijos, Lionel Leroux, Louis Labrousse, Alexandre Metras, Matthieu Michaud, Marie Hébert, Patricia Réant, Stéphane Lafitte

**Affiliations:** a CHU de Bordeaux, Service médico-chirurgical de valvulopathies et cardiomyopathies, chirurgie cardiaque adulte, cardiologie interventionnelle structurelle adulte, F-33000, Bordeaux, France; b Université de Bordeaux, CIC-P 1401, INSERM, Institut Bergonié, F-33000, Bordeaux, France; c Service de Cardiologie, Centre Hospitalier Universitaire de Sherbrooke, Sherbrooke, Québec, Canada

## Abstract

MitraClip (Abbott Laboratories, Abbott Park, IL) is validated in high-risk patients with severe degenerative mitral regurgitation (MR); however, it is not well established for functional MR in hypertrophic cardiomyopathy (HCM). We share a case of a 68-year-old man with HCM hospitalized for multiple incidents of acute pulmonary edema caused by a dynamic MR and successfully treated with the MitraClip device. Novel teaching points emerging from this case are that MRs in HCM can often be explained by mixed mechanisms, and properly identifying the MR mechanism is essential to choose optimal treatment. Furthermore, MitraClip can simultaneously treat MR secondarily to annular dilation and systolic anterior motion.

## Résumé

Il a été établi démque Le Mitraclip (Abbott Laboratories, Abbott Park, Il) est une intervention percutanée validée pour la prise en charge des patients à haut risque chirurgical qui présente une régurgitation mitrale (RM) sévère dégénérative. Toutefois, cette technique est moins bien établie dans une RM fonctionnelle dans le cadre d'une cardiomyopathie hypertrophique (CMH). Nous faisons part d'un cas d’un homme de 68 ans atteint d’une CMH et hospitalisé en raison de multiples œdèmes aigus du poumon causés une la RM dynamique dont le traitement par MitraClip s’est avéré une réussite. Les nouveaux d'enseignement à enseigner qui émergent de ce cas portent sur le fait que les RM dans le cadre d’une CMH s’expliquent souvent par des mécanismes mixtes et que la détermination exacte du mécanisme de la RM est essentielle pour choisir le traitement qui convient le mieux au patient. De plus, le MitraClip permet de traiter simultanément les deux mécanismes d'une RM due à une dilatation annulaire et au mouvement systolique antérieur.

Functional mitral regurgitation (MR), in which the valve leaflets are structurally normal, is explained by a left-atrioventricular anomaly, either by annular dilation or an alteration of the left-ventricular remodelling. The use of MitraClip (Abbott Laboratories, Abbott Park, IL) for the management of secondary MR in patients with prohibitive surgical risk is recognized as a class IIb indication in 2017 European Society of Cardiology (ESC) guidelines. The 2020 Focused Update of the 2017 American College of Cardiology (ACC) guidelines shows that the MitraClip device could be considered in carefully selected patients with secondary MR.^1^, ^2^ The percutaneous edge-to-edge valvular repair is safe and has been used in the management of functional MR in various clinical situations, other than heart failure with reduced ejection fraction, as studied in the Percutaneous Repair With the MitraClip Device for Severe Secondary Mitral Regurgitation (MITRA-FR) and **C**ardiovascular **O**utcomes **A**ssessment of the MitraClip **P**ercutaneous **T**herapy for Heart Failure Patients With Functional Mitral Regurgitation (COAPT).^3^ In 2014, the MitraClip device was used for the first time in obstructive hypertrophic cardiomyopathy (HCM) to treat systolic anterior motion (SAM) and the associated MR. In a small case series, the use of MitraClip showed a significant reduction in MR and left-ventricular outflow tract (LVOT) obstruction and has recently been used in the management of patients with obstructive HCM who are not candidates for septal reduction.^4^

We present an interesting case of a dynamic and unstable MR on annular dilation caused by left-atrial (LA) enlargement and a SAM causing LVOT obstruction and significant hemodynamic destabilization. The patient has been successfully treated with the MitraClip device.

## Case

**Figure 1:**
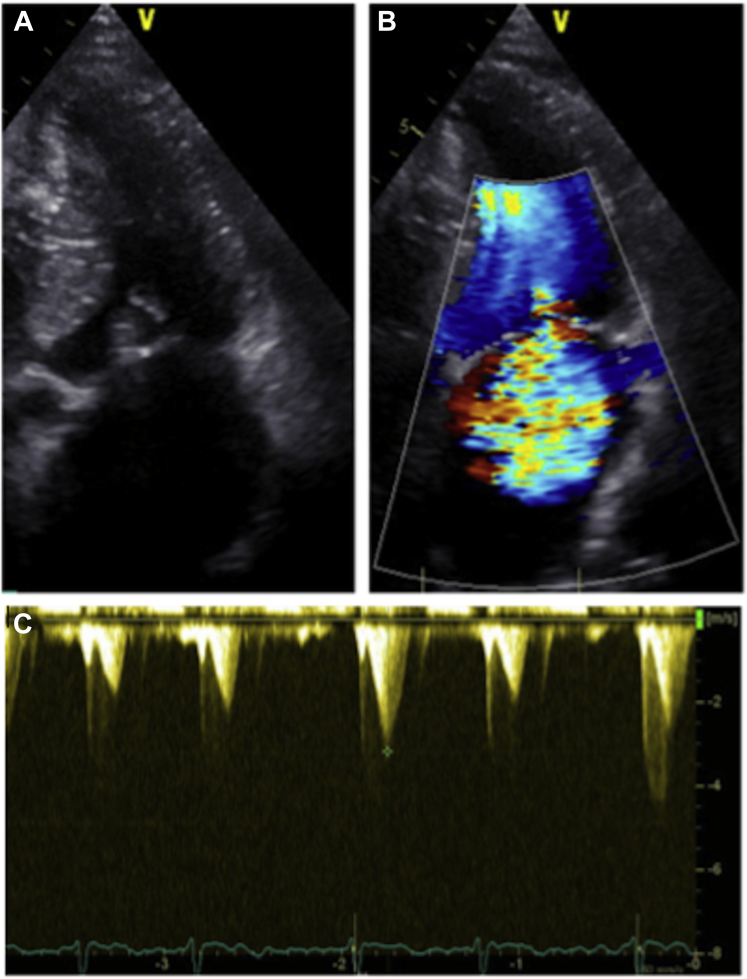
Transthoracic echocardiography showing a dilated left atrium associated with a systolic anterior motion of the mitral valve apparatus (**A**) and a severe central mitral regurgitation (**B**). Left-ventricular outflow tract obstruction driven by the systolic anterior motion (40 mm Hg) (**C**).

A 68-year-old man, known for HCM and nonsevere functional MR, suffered 2 incidents of acute pulmonary edema, following his atrial fibrillation ablation, in 2018 and 2019, the last of which was complicated by cardiogenic shock requiring transient mechanical support with an IMPELLA device (Abiomed, Danvers, MA). Transthoracic echocardiography (TTE) at that time showed a sudden deterioration of the MR, quantified as severe, with a centrally directed jet, possibly caused by procedural volume overload and the appearance of a SAM, causing the hemodynamic instability. The patient was stabilized and discharged with mild MR on the TTE. In January of 2020, the patient was hospitalized for another cardiac decompensation and transferred to our centre for evaluation. After diuretic treatment, transesophageal echocardiography (TEE) showed a mild-to-moderate central MR on an annular dilation (41 mm) caused by LA enlargement and no SAM. Despite optimized medical therapy, the patient had another fulminant cardiac decompensation in our centre that required respiratory support. A combination of a severe functional MR with a centrally directed jet and a subvalvular apparatus SAM causing LVOT obstruction was confirmed by TTE. The visualization of a central MR jet on the TTE is in favour of a severe functional MR caused by an annular dilation and not completely explained by the subvalvular apparatus SAM (Fig. 1).

**Figure 2:**
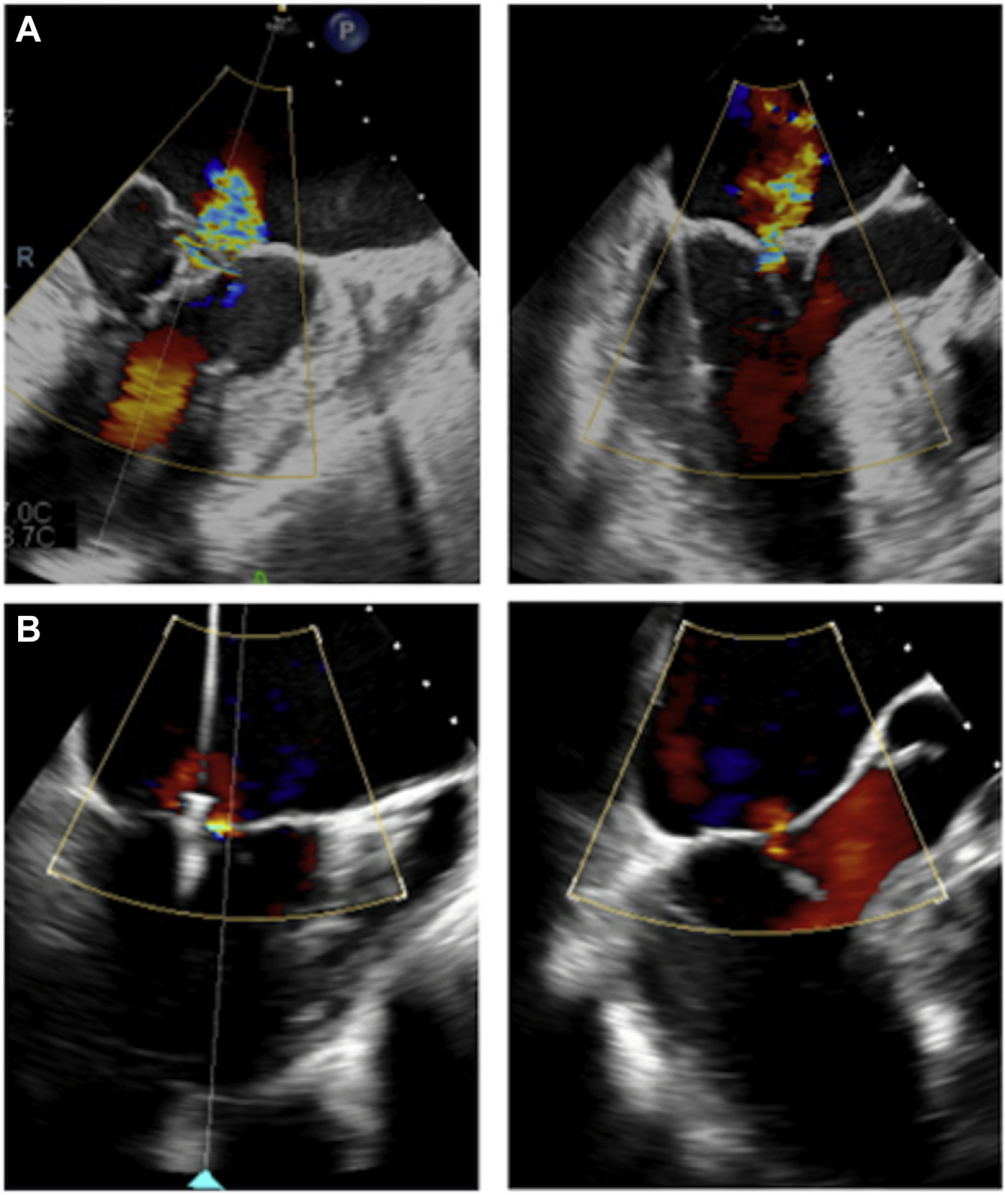
Transesophageal echocardiography X-plane views during MitraClip (Abbott Laboratories, Abbott Park, IL) procedure showing intermediate functional mitral regurgitation under general anesthesia (**A**). First clip installed in zone 2, leaving a residual eccentric lateral mitral regurgitation caused by a partial systolic anterior motion (**B**).

Facing this “eclipsed” MR and the fragility of the patient, the heart team estimated the risk of mortality or morbidity to be more than 15% and proposed the least invasive intervention. The unstable MR being explained by a mixed mechanism (annular dilation and a SAM), the team opted for a MitraClip procedure rather than an advanced surface ablation to treat both mechanisms at the same time. We proceeded with the installation of 2 NTR clips under general anaesthesia and 3-dimensional TEE guidance. The first clip was installed in zone 2 (A2-P2), leaving a moderate residual, posteriorly directed MR jet on an incomplete subvalvular apparatus SAM, where a second clip was installed (Fig. 2). The procedure was successful, with a postprocedural TTE showing mild residual MR. The patient's symptoms were significantly improved, and he was discharged to a rehabilitation centre. At 6-month follow-up, the patient has not been readmitted, and the TTE showed stable MitraClips with residual mild MR.

## Discussion

This case underlines how MR, caused by annular dilation, in which the coaptation length of the mitral valve leaflets is reduced, can become unstable rapidly under minimal hemodynamic change in afterload/preload and can lead to the appearance of a SAM, secondary to the tachycardia and the increased cardiac inotropy. LVOT obstruction can therefore lead the patient into profound cardiogenic shock. We emphasize that MR on annular dilatation in the HCM population seems to have a much less predictive evolution with a sometimes fulminant presentation. MRs in HCM are not all caused SAM and are often explained by mixed mechanisms.

**Novel Teaching Points**

• Properly identifying the initial MR mechanism is essential in choosing the optimal treatment.
• MitraClip can simultaneously treat MR on annular dilation and SAM.

## Funding Sources

The authors report no funding sources for this article.

## Disclosures

The authors have no conflicts of interest to disclose.
